# Supplementary material for: Long-Term LDL-Apheresis Treatment and Dynamics of Circulating miRNAs in Patients with Severe Familial Hypercholesterolemia
Source: Genes (Basel). 2023 Aug 1;14(8):1571. doi: 10.3390/genes14081571 (PMC10454435; doi:10.3390/genes14081571)

S Table S1 MiRNAs involved in atherosclerosis development

miRNA	Target genes	Functions	Process in development of atherosclerosis	References
**miR-181a**	TAB2; NEMO, Prox1	Decrease proinflammatory gene expression and the infiltration of macrophage, leukocyte and T cell into the lesions.	***Endothelial dysfunction***	*Su et al. 2019*
**miR-126**	SPRED-1; PIK3R; VCAM-1; CXCL12	Inhibits leukocyte adherence to ECs. Regulates angiogenic signaling and vascular integrity		*Asgeisdottir et a. 2012*
**miR-17-3p**	ICAM1	Decreases leukocytes adhesion to activated ECs		*Suarez et al. 2010*
**miR-92a**	KLF4/2	Regulates or contributes to flow-mediated EC activation and vascular inflammation		*Fang et al. 2012*
**miR-34**	SIRT1	EC senescence, suppresses cell proliferation		*Ito et al. 2010*
**miR-33a/b**	ABCA1, ABCG1, NPC1, CPT1A, SIRT6, AMPK, HADHB, CROT, CYP7A1, ABCB11, ATP8B1, NSF, SRC3, PCK1, G6PC, IRS2, RIP140, NFYC, SREBP1	Cholesterol efflux, HDL biogenesis, intracellular cholesterol transport, fatty acid oxidation, insulin signaling and glucose metabolism	***Cholesterol metabolism***	*Rayner et al. 2010*<br>*Rayner, Essau et al. 2011*<br>*Dávalos et al. 2011*<br>*Feinberg and Moore 2016 (reviewed)*
**miR-122**	SREBP, MTTP, Klf6	Fatty acid oxidation, cholesterol synthesis. Lipids accumulation in liver, fibrosis and tumor formation		*Essau et al. 2006*
**miR-370**	ABCA1; CPT1a, FASN, NR1H3, ACACA	Cholesterol efflux, HDL biogenesis, fatty acid oxidation		*Iliopoulos et al. 2010*
**miR27a**	RXRα, ABCA1, FASN, SREBP1/2, PPARα/γ, ApoA1, ApoB100, ApoE3	Lipid metabolism, cholesterol efflux		Shirasaki T et al. 2013
**miR185/342**	SREBP	Fatty acid oxidation, cholesterol metabolism		*Wang et al. 2013*
**miR-758**<br>**miR-144**	ABCA1	Cholesterol efflux		*Ramirez et al. 2011*<br>*Ramirez et al. 2013*
**mir-26a**	ABCA1; ARL7	Cholesterol efflux, HDL homeostasi		[a]*Sun et al. 2012*
**miR-155**	Bcl6, HBP1, AT1R; Ets-1; IL13 receptor α1)	Regulation of angiogenesis and inflammation	***Plaque development***	*Huang et al. 2010*
**miR-125a-5p**	LOX-1; CD68; SRB1, ORP9	Lipid uptake, cytokine expression, oxLDL internalization		*Chen et al. 2009*

**miR-29c**	ELN; COL1A; COL3A; Spry1	Vascular homeostasis and the posttranscriptional regulation of many extracellular matrix proteins		*Ulrich et al. 2016*
**miR-143/145**	Klf4, myocardin, Elk-1	Promote differentiation and repress proliferation of SMCs		*Lovren et al. 2012*
**miR-26a**	SMAD-1	Protecting VSMCs from differentiation and apoptosis		*Leeper et al. 2011*
**miR-155**	AT1R; Ets-1	Protective role in development of endothelial inflammation	***Neoangiogenesis***	[b]*Sun et al. 2012*
**miR-17-92**	ABCA1; LDLR; SEMA6A	Cholesterol efflux; promoting of angiogenesis by regulation of ECs repulsion		*Suarez et al. 2008*
**miR-27a/b**	ABCA1; SEMA6A			*Urbich et al. 2012*
**miR-146a/b**	TLR4; IRAK1; TRAF6	Lipid accumulation, inflammatory response	***Plaque rupture***	*Guo et al. 2010*
**miR-365**	Bcl2	ox-LDL-induced ECs apoptosis by regulating the expression of Bcl-2		*Qin et al. 2011*
**miR-21**	FABP7, PPARα, PTEN, Bcl-2, PDCD4	Depression of AP-1, inflammatory response, proliferation of SMCs, senescence		*Lin et al. 2009*

References:

42. Asgeirsdottir, S.A.; Van Solingen, C.; Murniati, N.F.; Zwiers, P.J.; Heeringa, P.; Van Meurs, M.; Satchell, S.C.; Saleem, M.A.; Mathieson, P.W.; Banas, B.; et al. Microrna-126 Contributes To Renal Macrovascular Heterogeneity Of Vcam-1 Protein Expression In Acute Inflammation. *Am J Physiol Renal Physiol* **2012,** *302*, F1630-F1639. doi: 10.1152/ajprenal.00400.2011.
43. Chen, T.; Huang, Z.; Wang, L.; Wang, Y.; Wu, F.; Meng, S.; Wang, C. MicroRNA-125a-5p partly regulates the inflammatory response, lipid uptake, and ORP9 expression in oxLDL-stimulated monocyte/macrophages. *Cardiovasc Res* **2009**, *83*, 131-139. doi: 10.1093/cvr/cvp121.
44. Dávalos, A.; Goedeke, L.; Smibert, P.; Ramírez, C.M.; Warrier, N.P.; Andreo, U.; Cirera-Salinas, D.; Rayner, K.; Suresh, U.; Pastor-Pareja, J.C.; et al. miR-33a/b contribute to the regulation of fatty acid metabolism and insulin signaling. *Proc Natl Acad Sci U S A.* **2011,** *108*(22), 9232-7. doi: 10.1073/pnas.1102281108.
45. Esau, C.; Davis, S.; Murray, S.F.; Yu, X.X.; Pandey, S.K.; Pear, M.; Watts, L.; Booten, S.L.; Graham, M.; McKay, R.; et al. miR-122 regulation of lipid metabolism revealed by in vivo antisense targeting. *Cell Metab*. **2006,** *3*(2):87-98. doi: 10.1016/j.cmet.2006.01.005.
46. Fang, Y.; Davies, P.F. Site-specific microRNA-92a regulation of Kruppel-like factors 4 and 2 in atherosusceptible endothelium. *Arterioscler Thromb Vasc Biol*. **2012,** *32(4)*, 979-987. doi: 10.1161/ATVBAHA.111.244053.
47. Feinberg, MW.; Moore, K.J. Microrna Regulation Of Atherosclerosis. *Circ Res* **2016,** *118***:** 703-720. doi: 10.1161/CIRCRESAHA.115.306300.
48. Guo, M.; Mao, X.; Ji, Q.; Lang, M.; Li, S.; Peng, Y.; Zhou, W.; Xiong, B.; Zeng, Q. miR-146a in PBMCs modulates Th1 function in patients with acute coronary syndrome. *Immunol Cell Biol*. **2010**, *88*, 555-564. doi: 10.1038/icb.2010.16.
49. Huang, R.S.; Hu, G.Q.; Lin, B.; Lin, Z.Y.; Sun, C.C. MicroRNA-155 silencing enhances inflammatory response and lipid uptake in oxidized low-density lipoprotein-stimulated human THP-1 macrophages. *J Invest Med.* **2010,** *58*, 961-967. doi: 10.231/JIM.0b013e3181ff46d7
50. Iliopoulos, D.; Drosatos, K.; Hiyama, Y.; Goldberg, .IJ.; Zannis, V.I. MicroRNA-370 controls the expression of microRNA-122 and Cpt1alpha and affects lipid metabolism. *J Lipid Res*. **2010,** *51*, 1513–1523. doi: 10.1194/jlr.M004812.
51. Ito, T.; Yagi, S.; Yamakuchi, M. Microrna-34a Regulation Of Endothelial Senescence. *Biochem Biophys Res Commun*, **2010**, *398*, 735-740. doi: 10.1016/j.bbrc.2010.07.012.

52. Leeper, N.J.; Raiesdana, A.; Kojima, Y.; Chun, H.J.; Azuma, J.; Maegdefessel, L.; Kundu, R.K.; Quertermous, T.; Tsao, P.S.; Spin, J.M. MicroRNA-26a is a novel regulator of vascular smooth muscle cell function. *J Cell Physiol* **2011**, *226*, 1035-1043. doi: 10.1002/jcp.22422.
53. Lin, Y.; Liu, X.; Cheng, Y.; Yang, J.; Huo, Y.; Zhang, C. Involvement of MicroRNAs in hydrogen peroxide-mediated gene regulation and cellular injury response in vascular smooth muscle cells. *J Biol Chem* **2009**, *284*, 7903-7913. doi: 10.1074/jbc.M806920200.
54. Lovren, F.; Pan, Y.; Quan, A.; Singh, K.K.; Shukla, P.C.; Gupta, N.; Steer, B.M.; Ingram, A.J.; Gupta, M.; Al-Omran, M. MicroRNA-145 targeted therapy reduces atherosclerosis. *Circulation.* **2012**, *126*, S81-S90. doi: 10.1161/CIRCULATIONAHA.111.084186.
55. Qin, B.; Xiao, B.; Liang, D.; Xia, J.; Li, Y.; Yang, H. MicroRNAs expression in ox-LDL treated HUVECs: MiR-365 modulates apoptosis and Bcl-2 expression. *Biochem Biophys Res Commun*. **2011**, *410*, 127–133. doi: 10.1016/j.bbrc.2011.05.118.
56. Ramirez, C.M.; Dávalos, A.; Goedeke, L.; Salerno, A.G.; Warrier, N. Cirera-Salinas, D.; Suárez, Y.; Fernández-Hernando C. MicroRNA-758 regulates cholesterol efflux through posttranscriptional repression of ATP-binding cassette transporter A1. *Arterioscler Thromb Vasc Biol.* **2011,** *31(11),* 2707-2714. doi: 10.1161/ATVBAHA.111.232066.
57. Ramirez, C.M.; Rotllan, N.; Vlassov, A.V.; Davalos, A.; Li, M.; Goedeke, L.; Aranda, J.F.; Cirera-Salinas, D.; Araldi, E.; Salerno, A.; et al. Control of cholesterol metabolism and plasma high density lipoprotein levels by microRNA-144. *Circ Res.* **2013**, *112*, 1592–1601. doi: 10.1161/CIRCRESAHA.112.300626.
58. Rayner, K.J.; Esau, C.C.; Hussain, F.N.; McDaniel, A.L.; Marshall, S.M.; van Gils, J.M.; Ray, T.D.; Sheedy, F.J.; Goedeke, L.; Liu, X.; et al. Inhibition of miR-33a/b in non-human primates raises plasma HDL and lowers VLDL triglycerides. *Nature.* **2011,** *478(7369),* 404-407. doi: 10.1038/nature10486.
59. Rayner, K.J.; Sheedy, F.J.; Esau, C.C.; Hussain, F.N.; Temel, R.E.; Parathath, S.; Van Gils, J.M.; Rayner, A.J.; Chang, A.N.; Suarez, Y. et al. Antagonism Of Mir-33 In Mice Promotes Reverse Cholesterol Transport And Regression Of Atherosclerosis. *J Clin Invest.* **2011**, *121*, 2921-2931. doi: 10.1172/JCI57275.
60. Shirasaki, T.; Honda, M.; Shimakami, T.; Horii, R.; Yamashita, T.; Sakai, Y.; Sakai, A.; Okada, H.; Watanabe, R.; Murakami, S. et al. MicroRNA-27a regulates lipid metabolism and inhibits hepatitis C virus replication in human hepatoma cells. *J Virol*. **2013**, *87(9)*, 5270-5286. doi: 10.1128/JVI.03022-12.
61. Su, Y.; Yuan, J.; Zhang, F.; Lei, Q.; Zhang, T.; Li, K.; Guo, J.; Hong, Y.; Bu, G.; Lv, X. et al. MicroRNA-181a-5p and microRNA-181a-3p cooperatively restrict vascular inflammation and atherosclerosis. *Cell Death Dis.* **2019**, *10(5)*, 365. doi: 10.1038/s41419-019-1599-9.
62. Suarez, Y.; Fernández-Hernando, C.; Yu, J.; Gerber, S. A.; Harrison, K.D.; Pober, J.S.; Iruela-Arispe, M.L.; Merkenschlager, M.; Sessa, W.C. Dicer-dependent endothelial microRNAs are necessary for postnatal angiogenesis. *Proc Natl Acad Sci USA.* 2008, *105,* 14082-14087. doi: 10.1073/pnas.0804597105.
63. Suárez, Y.; Wang, C.; Manes, T.D.; Pober, J.S. Cutting edge: TNF-induced microRNAs regulate TNF-induced expression of E-selectin and intercellular adhesion molecule-1 on human endothelial cells: feedback control of inflammation. *J Immunol*. **2010** *184(1),* 21-25. doi: 10.4049/jimmunol.0902369.
64. [a]Sun D, Zhang J, Xie J,WeiW, Chen M, Zhao X. MiR-26 controls LXR-dependent cholesterol efflux by targeting ABCA1 and ARL7. *FEBS Lett*, **2012**, *586*, 1472–1479. doi: 10.1016/j.febslet.2012.03.068.
65. [b]Sun, H.X.; Zeng, D.X.; Li, R.T.; Pang, R.P.; Yang, H.; Hu, Y.L.; Zhang, Q.; Jiang ,Y.; Huang, L.Y.; Tang, Y.B. et al. Essential role of microRNA-155 in regulating endothelium dependent vasorelaxation by targeting endothelial nitric oxide synthase. *Hypertension.* **2012**, *60*, 1407-1414. doi: 10.1161/HYPERTENSIONAHA.112.197301.
66. Ulrich, V.; Rotllan, N.; Araldi, E.; Luciano, A.; Skroblin, P.; Abonnenc, M.; Perrotta, P.; Yin, X.; Bauer, A.; Leslie, K.L. et al. Chronic miR-29 antagonism promotes favorable plaque remodeling in atherosclerotic mice. *EMBO Mol Med*. **2016**, *8(6*), 643-653. doi: 10.15252/emmm.201506031.
67. Urbich, C.; Kaluza, D.; Frömel, T.; Knau, A.; Bennewitz, K.; Boon, R.A.; Bonauer, A.; Doebele, C.; Boeckel, J.N.; Hergenreider, E. et al. MicroRNA-27a/b controls endothelial cell repulsion and angiogenesis by targeting semaphorin 6A. *Blood*. **2012,** *119(6),* 1607-1616. doi: 10.1182/blood-2011-08-373886.
68. Wang, L.; Jia, X.J.; Jiang, H.J.; Du, Y.; Yang, F.; Si, S.Y.; Hong, B. MicroRNAs 185, 96, and 223 repress selective high-density lipoprotein cholesterol uptake through posttranscriptional inhibition. *Mol Cell Biol.* **2013**, *33*, 1956–1964. doi: 10.1128/MCB.01580-12.

S Table S2 Identification of measured miRNA assays and their mature miRNA sequences

miRBase ID	miRBase Accession	Mature miRNA sequence
hsa-miR-126-3p	MIMAT0000445	5´UCGUACCGUGAGUAAUAAUGCG
hsa-miR-155-5p	MIMAT0000646	5´UUAAUGCUAAUCGUGAUAGGGGUU
hsa-miR-122-5p	MIMAT0000421	5´UGGAGUGUGACAAUGGUGUUUG
hsa-miR-125a-5p	MIMAT0000443	5´UCCCUGAGACCCUUUAACCUGUGA
hsa-miR-146a-5p	MIMAT0000449	5´UGAGAACUGAAUUCCAUGGGUU
hsa-miR-181a-5p	MIMAT0000256	5´AACAUUCAACGCUGUCGGUGAGU
hsa-miR-17-5p	MIMAT0003326	5´AGGCGGGGCGCCGCGGGACCGC
hsa-miR-34a-5p	MIMAT0000255	5´UGGCAGUGUCUUAGCUGGUUGU
hsa-miR-33a-5p	MIMAT0000091	5´GUGCAUUGUAGUUGCAUUGCA
hsa-miR-370-3p	MIMAT0000722	5´GCCUGCUGGGGUGGAACCUGGU
hsa-miR-758-3p	MIMAT0003879	5´UUUGUGACCUGGUCCACUAACC
hsa-miR-144-5p	MIMAT0004600	5´GGAUAUCAUCAUAUACUGUAAG
hsa-miR-26a-5p	MIMAT0000082	5´UUCAAGUAAUCCAGGAUAGGCU
hsa-miR-29c-3p	MIMAT0000681	5´UAGCACCAUUUGAAAUCGGUUA
hsa-miR-143-5p	MIMAT0004599	5´GGUGCAGUGCUGCAUCUCUGGU
hsa-miR-92a-3p	MIMAT0000092	5´UAUUGCACUUGUCCCGGCCUGU
hsa-miR-27a-3p	MIMAT0000084	5´UUCACAGUGGCUAAGUUCCGC
hsa-miR-365a-3p	MIMAT0000710	5´UAAUGCCCCUAAAAAUCCUUAU
hsa-miR-185-5p	MIMAT0000455	5´UGGAGAGAAAGGCAGUUCCUGA
hsa-miR-21-5p	MIMAT0000076	5´UAGCUUAUCAGACUGAUGUUGA
hsa-miR-103-3p	MIMAT0000101	5'AGCAGCAUUGUACAGGGCUAUGA
hsa-miR-191-5p	MIMAT0000440	5'CAACGGAAUCCCAAAAGCAGCUG
hsa-let-7a-5p	MIMAT0000062	5'UGAGGUAGUAGGUUGUAUAGUU

S Table S3 Biomarkers of FH patients during time points of apheresis treatment

	FU1	FU2	FU3	FU4	FU5	FU6	FU7	FU8	P value
Total cholesterol (mmol/L)	5.2±1.3	5.5±1.6	5.3±1.6	4.3(3.3)	4.6±0.8	4.3(1.1)	4.6±1.2	4.1(4.6)	0.90
LDL cholesterol (mmol/L)	3.2±1.3	3.1±2.0	2.6±1.3	3.5±2.0	2.8±0.9	2.5(1.6)	2.8±1.3	1.8 (4.1)	0.95
HDL cholesterol (mmol/L)	1.3±0.4	1.4(1.2)	1.3(1.5)	1.3±0.5	1.2±0.5	1.2(0.6)	1.2±0.3	1.1(0.7)	0.58
Triacylglycerol (mmol/L)	1.3(1.9)	1.4(2.7)	1.4(1.3)	1.3(1.8)	2.3±1.3	2.3±1.6	1.8±1.0	1.6(4.1)	0.89
Glycemia (mmol/L)	/	5.7(4.8)	5.4(4.4)	5.8(4.4)	7.2(7.3)	6.2(2.8)	6.9±2.6	6.2(6.3)	0.88
ApoB (g/L)	/	1.3±0.4	1.3±0.4	1.2±0.4	1.1±0.3	1.1(0.3)	1.1±0.4	1.1(1.2)	0.90
Lp(a) (nmol/L)	/	18.3(38.2)	28.3(128.2)	24.2(80.7)	12.5(52.8)	13.1(203.5)	26.5(244.2)	11.4(38.2)	0.91
ALT (µkat/L)	/	0.5(0.4)	0.5±0.3	0.5(0.6)	0.4(0.4)	0.5±0.3	0.5±0.3	0.9(0.9)	0.84
AST (µkat/L)	/	0.6±0.2	0.5±0.2	0.4(0.4)	0.4(0.3)	0.4(0.2)	0.5±0.2	0.6(0.6)	0.94
Creatinine (µmol/L)	/	2.9±1.7	2.7±1.7	2.7±1.6	2.6±1.6	3.2±2.4	2.5±1.3	2.2(8.3)	0.99

Data are shown as the mean ± SD or as median and (IQR). /- depicts that data are not available. P-value depicts differences during FU time. ALT-alanine aminotransferase, ApoB-apolipoprotein B, Lp(a)-lipoprotein (a), AST-aspartate aminotransferase, HDL-high density lipoprotein, LDL-low density lipoprotein.

S Figure S1 Overall treatment time effect on variances in levels of pro-inflammatory miR-146a. Group 1: patients with mean overall treatment times of 17.9±4.3 years (N=6) and Group 2: patients with mean overall treatment times 5.5±3.3 years (N=6). Bar graphs demonstrate the mean ± standard error.

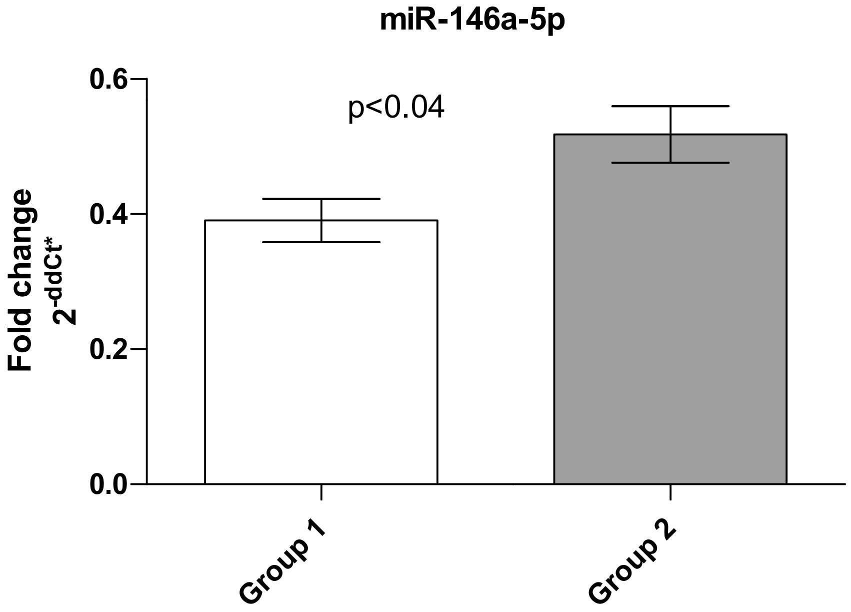

S Figure S2 Comparison of miRNAs plasma concentration during FUs in respect to absorption technique: rheopheresis (red line) vs. LDL apheresis (blue line) in FU time points (1-8). Data are expressed as means ± standard error. All p = n.s.

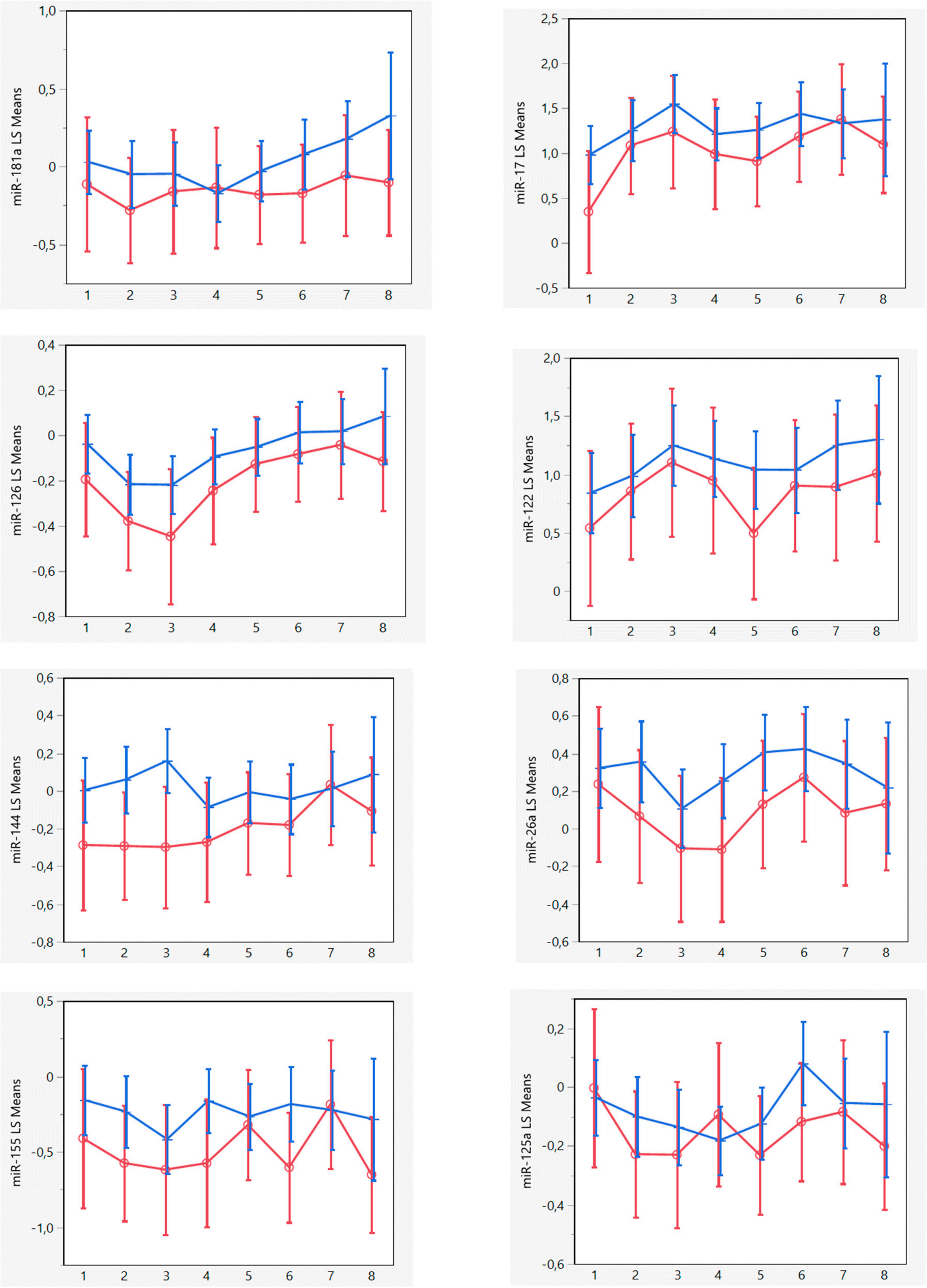

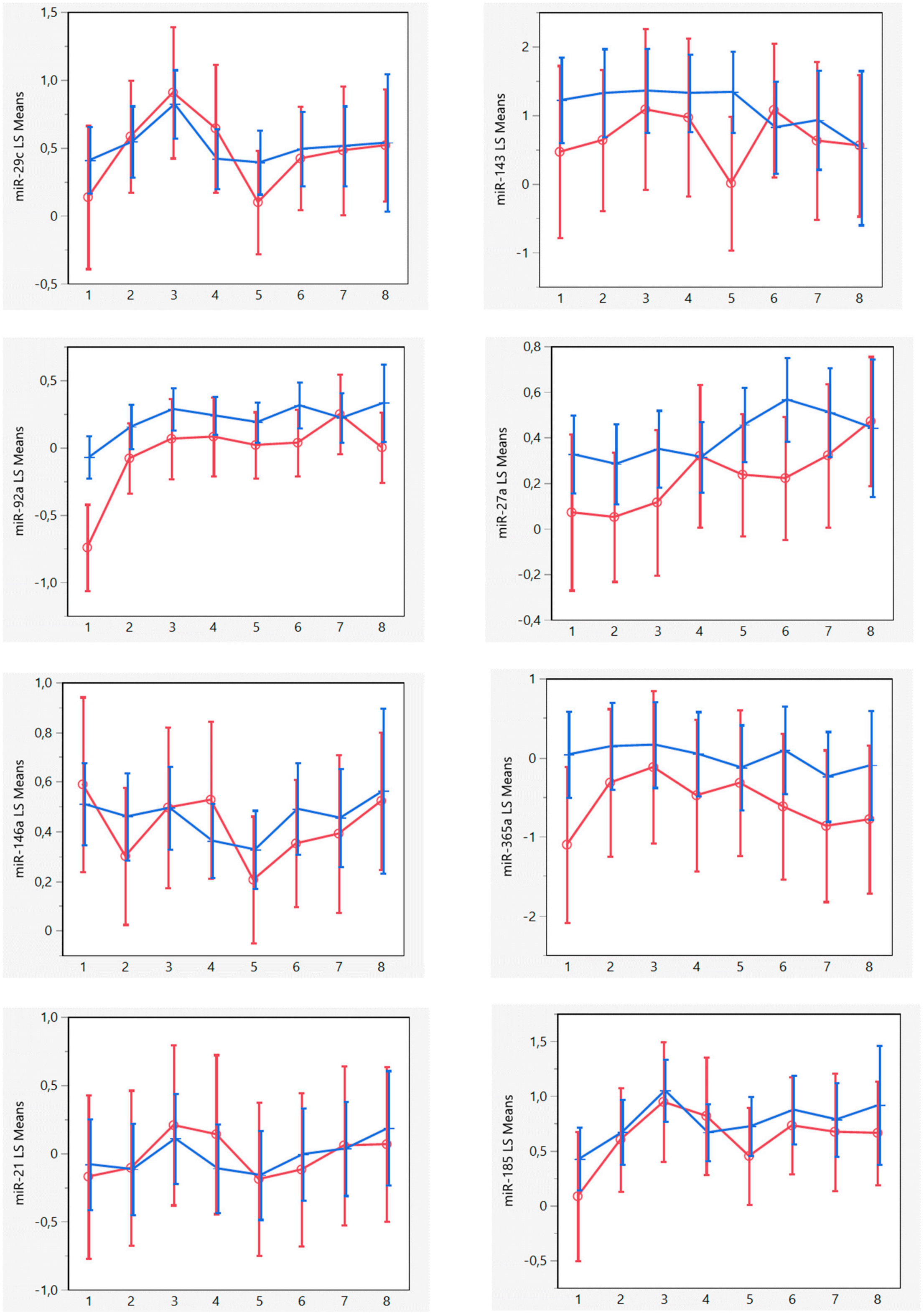
miR-29c LS Means
1,5
1,0
0,5
0
-0,5
1 2 3 4 5 6 7 8
miR-143 LS Means
2
1
0
-1
1 2 3 4 5 6 7 8
miR-92a LS Means
0,5
0
-0,5
-1,0
1 2 3 4 5 6 7 8
miR-27a LS Means
0,8
0,6
0,4
0,2
0
-0,2
-0,4
1 2 3 4 5 6 7 8
miR-146a LS Means
1,0
0,8
0,6
0,4
0,2
0
1 2 3 4 5 6 7 8
miR-365a LS Means
1
0
-1
-2
1 2 3 4 5 6 7 8
miR-21 LS Means
1,0
0,5
0
-0,5
-1,0
1 2 3 4 5 6 7 8
miR-185 LS Means
1,5
1,0
0,5
0
-0,5
1 2 3 4 5 6 7 8

S Figure S3 Differences in miRNAs levels between patients with type 2 diabetes mellitus (T2DM) vs. non diabetes. diabetes (blue line) vs. non-diabetes (red line) in FU time points (1-8). Data are expressed as means ± standard error. For miR-155 is $p \leq 0.01$; for others all p = n.s.

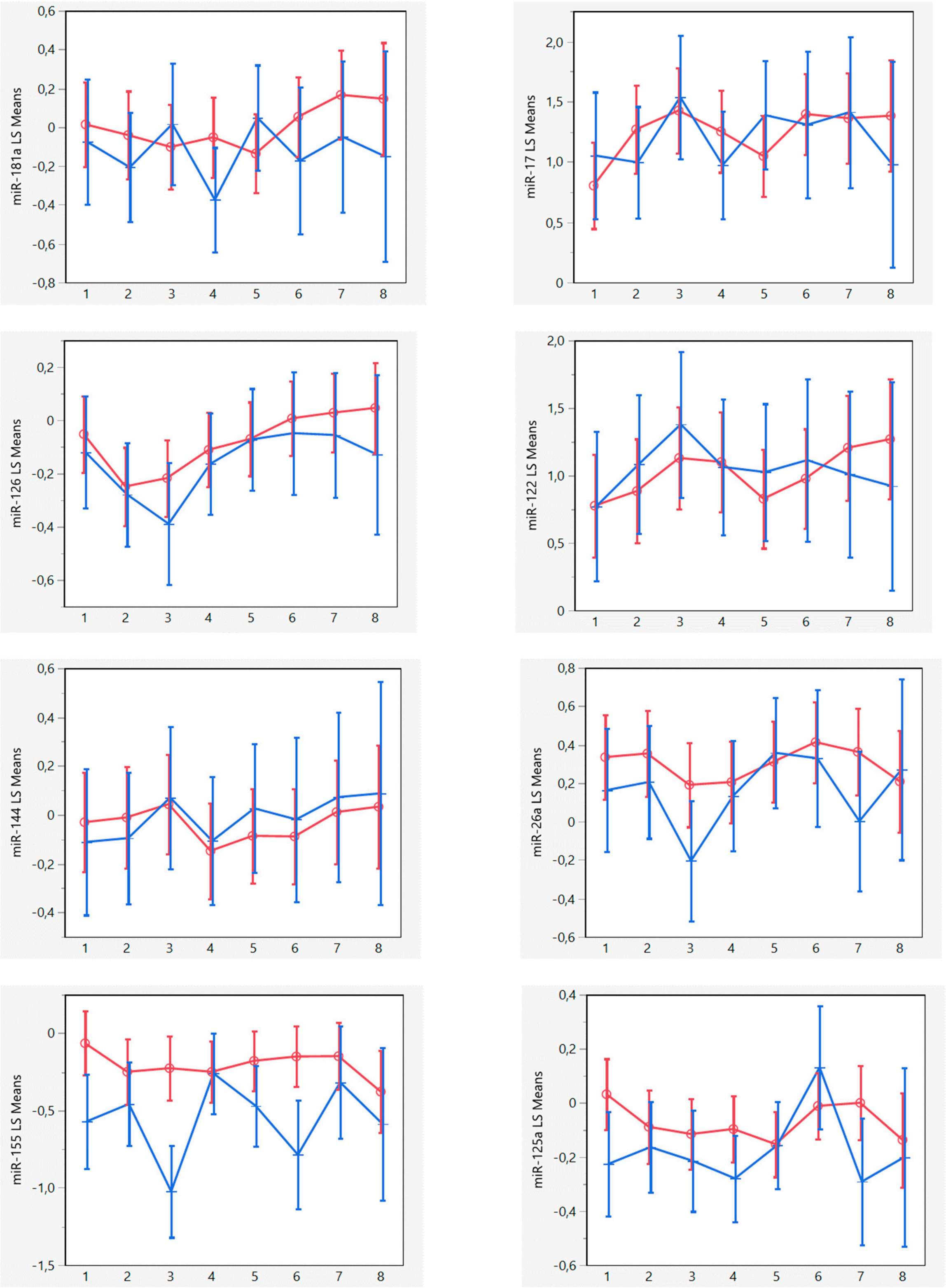

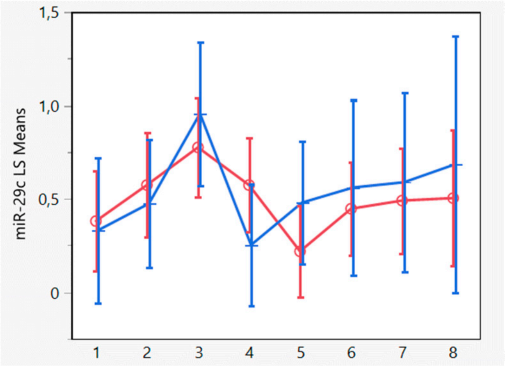
miR-29c LS Means
1,5
1,0
0,5
0
1 2 3 4 5 6 7 8

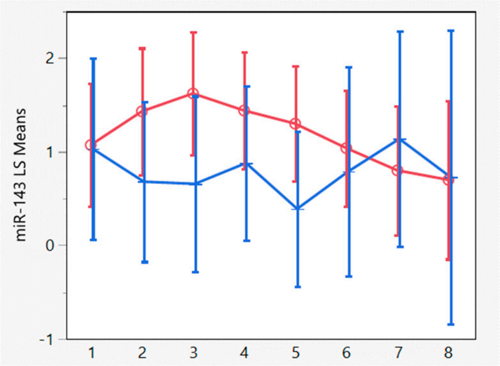
miR-143 LS Means
2
1
0
-1
1 2 3 4 5 6 7 8

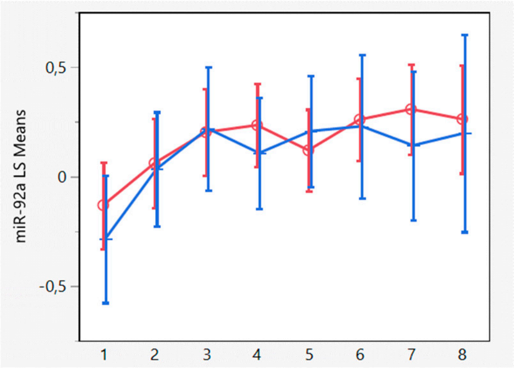
miR-92a LS Means
0,5
0
-0,5
1 2 3 4 5 6 7 8

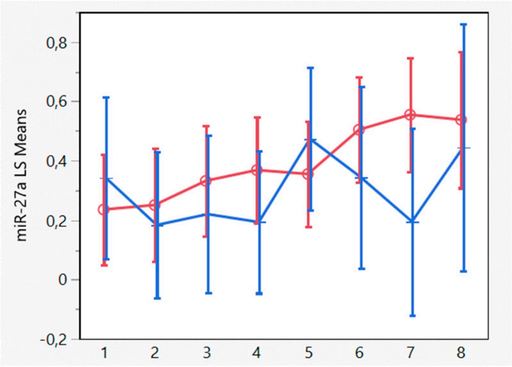
miR-27a LS Means
0,8
0,6
0,4
0,2
0
-0,2
1 2 3 4 5 6 7 8

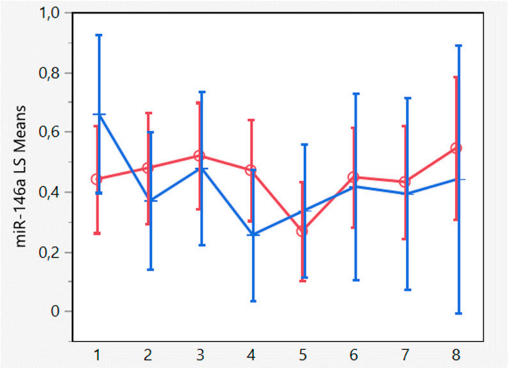
miR-146a LS Means
1,0
0,8
0,6
0,4
0,2
0
1 2 3 4 5 6 7 8

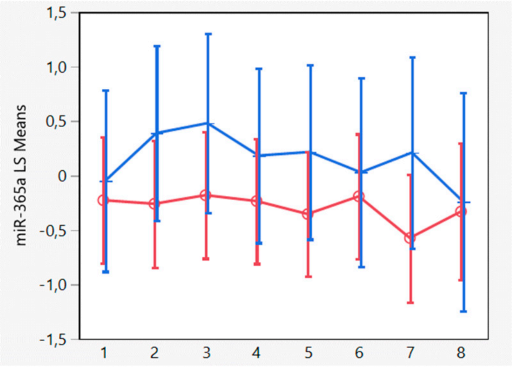
miR-365a LS Means
1,5
1,0
0,5
0
-0,5
-1,0
-1,5
1 2 3 4 5 6 7 8

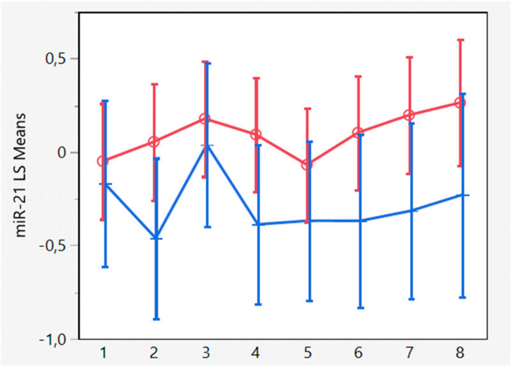
miR-21 LS Means
0,5
0
-0,5
-1,0
1 2 3 4 5 6 7 8

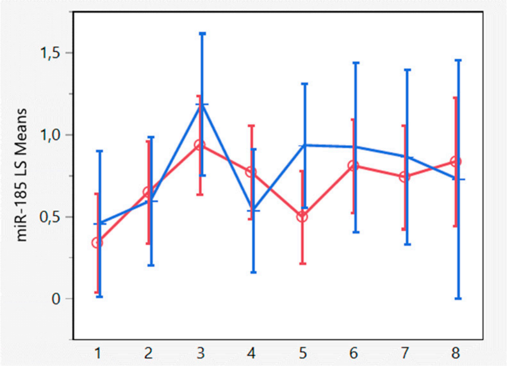
miR-185 LS Means
1,5
1,0
0,5
0
1 2 3 4 5 6 7 8

S Figure S4 Differences in plasma concentrations of LDL-C, TAG, and glycemia parameters between LDLR mutations

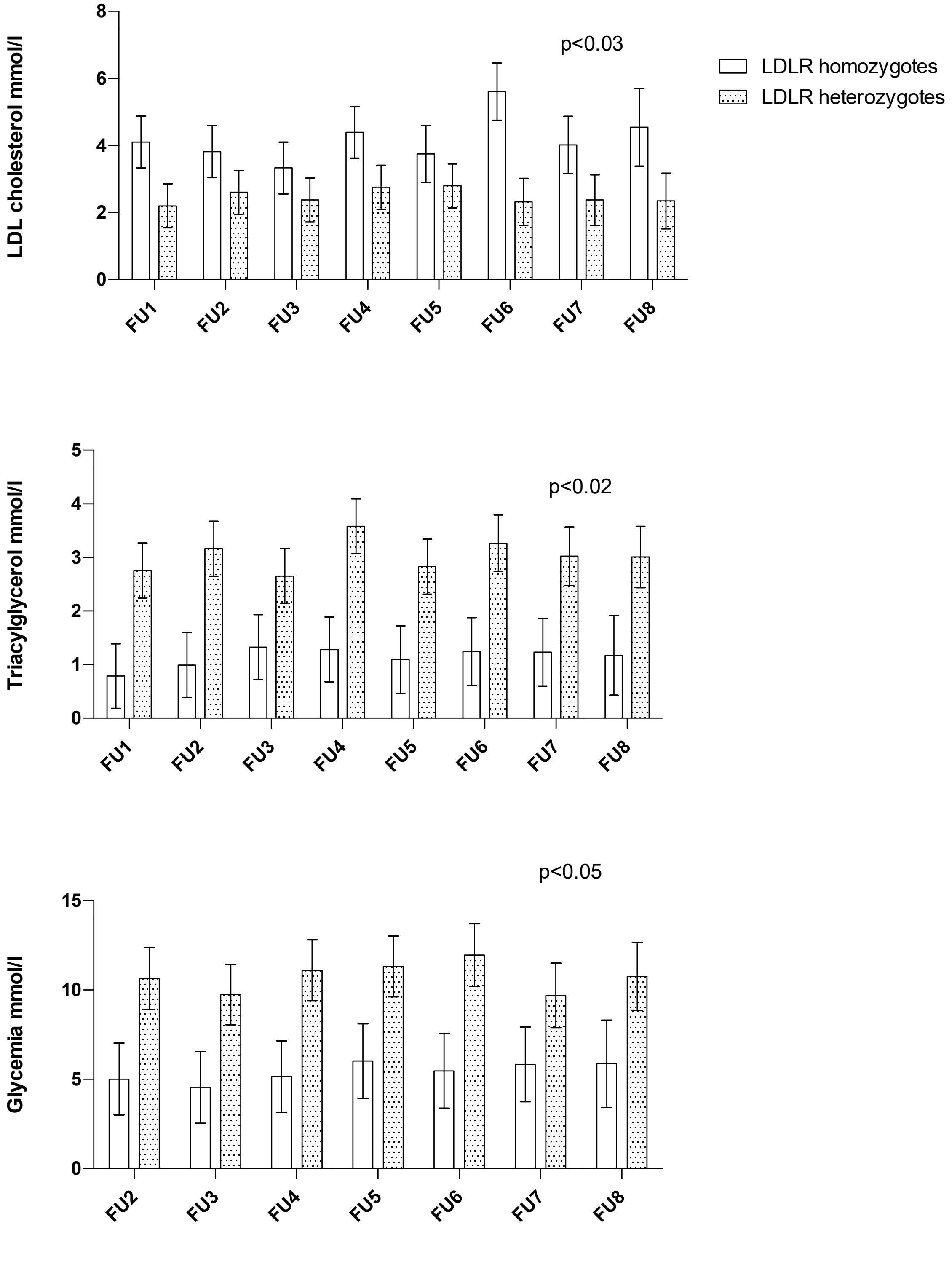

Supplement: Supplementary file 1 [file genes-14-01571-s001.zip › genes-2508047-supplementary.pdf]
